# Mr. Albert G. Brewster

**Published:** 1854-04

**Authors:** 


Obituary.—Died in Ossipee, N. H., in January, 1854, Mr. Albert G. Brewster, Surgeon Dentist, Æ. 30. Mr. Brewster departed this life in the mansion of his late father, John Brewster, Esq. He had been in the dental profession about seven years, and located during much of that time in the city of Salem, Mass. He was a descendant of William Brewster the elder, one of the Plymouth Rock Pilgrims, by the branch of Wrestling Brewster, the elder's third son, and of the seventh generation of descendants.

